# Stories from the Greenhouse—A Brief on Cotton Seed Germination

**DOI:** 10.3390/plants10122807

**Published:** 2021-12-18

**Authors:** Andrea B. Maeda, Leslie W. Wells, Monica A. Sheehan, Jane K. Dever

**Affiliations:** Texas A&M AgriLife Research and Extension Center, Lubbock, TX 79403, USA; leslie.wells@ag.tamu.edu (L.W.W.); mabellow@ag.tamu.edu (M.A.S.); jdever@ag.tamu.edu (J.K.D.)

**Keywords:** germination, cotton, plant research

## Abstract

Seed germination is the basis for the proliferation of sexual-reproducing plants, efficient crop production, and a successful crop improvement research program. Cotton (*Gossypium* spp.), the subject of this review, can be often sensitive to germination conditions. The hardness of the cotton seed coat, storage, extreme temperatures, and dormancy are some of the factors that can influence cotton seed germination. Research programs conducting studies on exotic and wild cotton species are especially affected by those hurdles. Here, we briefly review the challenges of cotton seed germination and some of the approaches our cotton breeding program explored throughout the years.

## 1. Introduction

From requests for germinating exotic cotton species seeds for botanical illustrations to germinating heritage seeds from wild cotton plants found in an abandoned cattle ranch on a Caribbean Island, our lab have seen and tried unusual methods to germinate cotton seed from all over the world. This is a compilation of our experiences and methodologies to germinate cotton seeds.

The importance of seeds and seed germination [1] dates from pre-agricultural times when humans uncovered the fascinating science of perpetuation of plants. For sexually reproducing species, mature planted seeds will germinate and become a new plant. This finding led to the beginning of a new era of plant domestication and cultivation, enabling mankind to settle and evolve—but what if seeds do not germinate?

While seeds are a vital form of dispersal and propagation of plants in the environment, they had to develop mechanisms in unfavorable circumstances to ensure the preservation of the next generation. One of the major survival adaptations of seeds is dormancy. This phenomenon allows the seed to delay and coordinate germination according to environmental conditions and it is essential to the conservation and proliferation of a species [2,3,4,5]. Generally, dormant seeds naturally start or resume the germination process once they detect environmental signals indicating suitable conditions for plant development such as temperature fluctuations, water availability, and even smoke [6,7]. If unfavorable conditions persist and seeds halt germination for a long period, aging might negatively impact this process. Modifications in the seed structure like the hard seed trait in cotton can also affect germination. Often referred to as physical dormancy, hard seeds are water impermeable and prevent gas exchange and water uptake by the embryo [8,9].

### 1.1. Cotton

Cotton (*Gossypium* spp.) has been cultivated in various regions of the world since prehistoric times. It was since domesticated from wild perennial plants to a herbaceous annual crop [10]. There are approximately 50 species in the genus *Gossypium*, some being tetraploids (2n = 4x = 52) and the majority diploids (2n = 2x = 26) [11]. There are four domesticated species of cotton, *Gossypium arboreum* (diploid), *Gossypium herbaceum* (diploid), *Gossypium hirsutum* (tetraploid), and *Gossypium barbadense* (tetraploid) [12]. *G. hirsutum* (also known as upland cotton) and *G. barbadense* (also known as pima cotton) are the most cultivated cotton species in the world. Domesticated cotton is unique among other modern cultivated major agronomic crops because of its perennial growth and indeterminate fruiting habit. Despite this singularity, cotton is grown as an annual crop often with the use of growth regulators and chemical, temperature, or mechanical termination. Another unique characteristic of cotton is its seed. Both maternal and filial tissues have economic value, since part of the seed coat epidermal cells grow into cellulose-rich fibers while the embryo cells produce protein and oils [13,14]. Because of these features, cotton is one of the most important crops in the world, grown predominantly for the fiber as the primary natural source of the world’s textile industry, and its seed is extensively used for feedstock and oil for human consumption [15,16].

### 1.2. Cotton Seed Anatomy and Physiology

**Figure 1:**
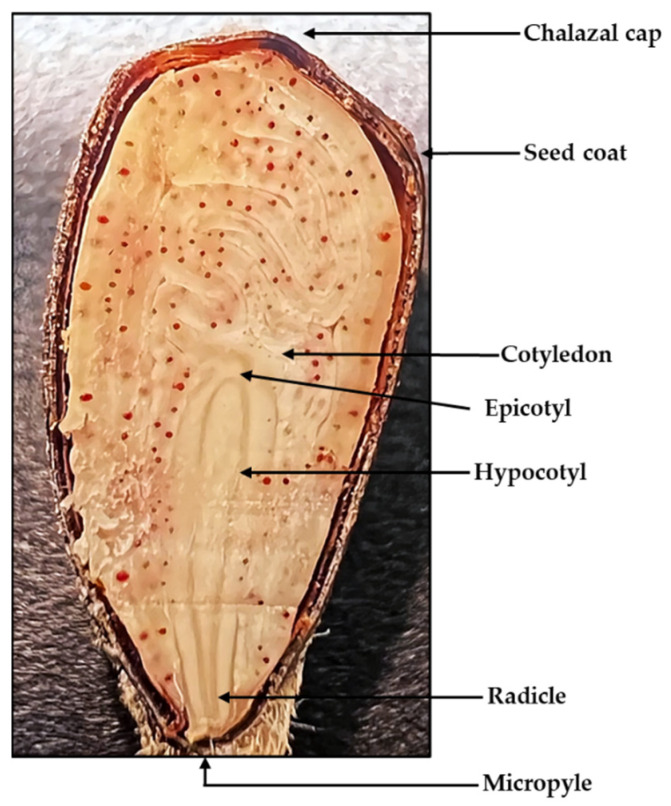
Anatomy of a cotton seed embryo.

The embryo in a mature cotton seed contains the radicle, hypocotyl, a primordial epicotyl, and two cotyledons (Figure 1). The cotyledons are the source of energy for germination and early seedling development. Under normal environmental conditions, the cotton seed starts the imbibition process through the chalazal cap and water moves through capillary action to the opposite, pointed side of the seed—the micropyle. This diffusive water intake softens the seed coat as well as builds a moisture reserve for the embryo in case water becomes scarce during germination. As water is absorbed, the seed swells causing a rupture on the seed coat at the micropylar end. The embryonic axis elongates, the radicle then emerges through the micropyle and grows downward, later becoming the taproot of the plant. During this process, oxygen is also absorbed through the seed coat initiating respiration. Under favorable conditions, the seed will enter full metabolic activity and a new cotton plant will develop [17,18,19,20,21,22]. Plant vigor and the successful establishment of a new individual are largely dependent on those primordial physiological and biochemical processes occurring in the seed. A disruption of any of those metabolic activities could hinder or entirely prevent the germination process, curtailing seedling growth.

### 1.3. Factors Affecting Cotton Seed Germination

With the advent of the Industrial Revolution, cotton became a major field crop and has since overcome many production challenges [23]. From the invention of the cotton gin to efficiently remove fiber from seed in the 1700s which relieved a burden in the mechanical processing and export of upland cotton [24], to the improvement of methods of delinting seed to avoid seed diseases and facilitate the use of a mechanical cotton planter [25,26,27], cotton has proven to be a complex crop that requires unique approaches in- and after season [28]. Moreover, in the early 1900s, Toole and Drummond [29] described cotton seeds as being “more sensitive to conditions of germination than the seed of most field crops”. Temperature, soil moisture, storage condition, seed moisture content, permeability of the seed coat, all can affect germination. Throughout the years, cotton seed quality and tolerance to environmental stresses improved with the development of new varieties, seed treatments, and breeding techniques, and so did field germination rates [21]. Under appropriate planting conditions, cotton field stands became more consistent and uniform. Nevertheless, despite advancements in commercial fields, cotton seed germination can still be an obstacle in laboratory and greenhouse studies. Crop improvement research facilities often work with old cotton varieties and wild species of cotton, especially when looking for genetic variation within the genus *Gossypium*. Considering that seed germination is a major component when doing research in plants, all the potential genetic resources available to plant scientists today could not be fully utilized without an adequate supply of quality seed. This paper will focus on cotton seed germination challenges in the context of small-scale research.

#### 1.3.1. Seed Coat

The integrity of the seed coat is an important attribute to maintain seed quality until it is time to plant. However, certain anatomical structure conditions may interfere with the physiological activities necessary for germination. A coat that is too hard with a lignified chalazal cap, also called “hard-seed”, could constrain the embryo and reduce water absorption, preventing germination [21,30,31,32]. This trait is uncommon in modern cotton varieties due to selections and breeding but is often seen in wild-type species. Seed coat water impermeability is often classified as physical dormancy, and it is caused by impermeable layers of palisade cells [8,33,34]. Christiansen and Moore [30] conducted microscopic studies to compare the structure of the cotton seed coats of hard seeds versus non-hard seeds. They found that in hard seeds, the structures near the chalazal area are highly compacted in comparison to the other seeds. There are some studies on different techniques to increase seed coat permeability and water absorption to boost cotton seed germination in a research environment. For example, soaking cotton seeds in hot water (80 °C to 85 °C) for at least one minute was found to improve the germination of dormant hard seeds [30,35]. Scarification of the seed coat is another approach common in many species that have water impermeable seeds [31,36]. Mechanical scarification, an abrasion method that removes part of the seed coat, is often used in legume crops [36]. The intensity of scarification can be regulated in commercial scarifiers to avoid more than necessary damage to the seed, and consequent injuries to the embryo. A study of different mechanical scarification treatments in cicer milkvetch (*Astralagus cicer*) reported greater germination in scarified seeds, although field stands were affected in treatments where seeds were “over-scarified” [37]. Mechanical scarification of cotton seed using a commercial seed scarifier has been attempted [27], but field emergence and establishment were affected. However, a small rupture on the cotton seed coat has been reported to aid in the germination of hard-seeded species by allowing the seed to absorb water efficiently [30,38]. Acid delinting is another known scarification method for expediting germination in several plant species, including cotton [39]. Marani and Amirav [40] reported improved and quicker germination of cotton at low temperatures after acid delinting. In more recent germination experiments, melatonin seed treatment has been reported to regulate the opening of stomata in the seed coat increasing germination rates in cotton seeds [41]. Studies on cold plasm treatment and its mechanisms that lead to a biological response in seeds showed an increase in seed water absorption, improved warm germination, and chilling tolerance in cotton [42,43].

#### 1.3.2. Drought 

Extreme water shortages can significantly constrain germination and seedling establishment of most field crops [44]. Although cotton is relatively tolerant to drought compared to other agricultural crops, marginal soil moisture can decrease germination rates. The process of imbibition that initiates germination is contingent on the water potential difference between the seed and the soil [45]. If water is scarce, internal seed moisture tension is weakened and water uptake may decrease. Moreover, hydration rates can be affected by other environmental conditions such as low temperatures, and those can vary among cotton species [17]. In one study, *Gossypium hirsutum* was found to have a lower hydration rate than *Gossypium barbadense* when seeds were subjected to various levels of chilling [46]. Drought-tolerant species and varieties, among other characteristics, may have developed mechanisms that facilitate seed water imbibition. These can include adaptations in the seed coat like increased water permeability, quick water intake, and different amounts of water requirements for germination. Stiles [47] reported seed adaptability in low water environments in drought-tolerant cotton and corn varieties. For those reasons, in greenhouse studies when different cotton species and seed sources are often used, it is important to consider that standardizing water treatments may result in variable seed germination responses.

#### 1.3.3. Temperature

Chilling temperatures can injure germinating cotton seeds, especially during the first stages of imbibition when seeds start soaking up water and are at their most sensitive phase [17,48]. Cold soil temperatures during this period can ultimately kill the embryo or cause long-term adverse consequences to the growing plant, like undeveloped tap root, delayed maturity, and ultimately lower yields [49,50,51]. Krzyzanowski and Delouche [52] used different lots of a commercial *G. hirsutum* cotton variety to report an optimal germination temperature of 28 °C to 30 °C, with significantly lower germination percentages when the temperature dropped below 20 °C. Ludwig [53] in 1932 indicated that upland cotton germination would be unlikely in temperatures lower than 11 °C.

#### 1.3.4. Seed Storage

Germination potential peak usually occurs shortly after harvest and decreases exponentially with long-term storage time. A decline in seed vigor and viability may be caused by a variety of biochemical processes in the seed that can be accelerated under inappropriate temperature and moisture conditions [54,55,56,57,58,59]. Most seed species preserve their viability best when dried and stored in low moisture content. However, according to an early 1900s study, cotton seeds that are stored in extremely dry conditions and seed moisture content falls below 6%, have an increased chance of becoming hard-seeded and germination will be affected. According to the authors, an approximate 12% moisture content in the seed is necessary for vigorous germination [29]. Low-temperature cold storage (0–5 °C) is also desirable for the retention of seed quality [20]. Therefore, proper seed storage conditions are essential to maintain viable seed stock in a research program.

#### 1.3.5. Dormancy

Dormancy can be caused by numerous factors and manifested in seasonal cycles according to environmental determinants; it is generally defined as the failure of a viable seed to germinate under favorable conditions [22,60]. There are two widely accepted distinctions in the dormancy cycle—primary dormancy and secondary dormancy. The prevention of germination in freshly harvested mature seeds caused by abscisic acid during seed development is denominated in primary dormancy [61,62]. This usually requires a combination of events to reinstate full metabolic activity, such as changes in temperature, moisture variation, or simply the passage of time [63]. Nevertheless, dispersed seeds could also enter a second state of dormancy when environmental factors are not as propitious to germination, also known as secondary dormancy [60]. According to Bewley and Black [20], mature seeds can enter a dormant state after imbibition and can survive fully imbibed for a long period of time. Taylor and Lankford [64] reported secondary dormancy in cotton under low temperature and high salinity stress in the 1970s.

In modern agricultural crops, due to primary dormancy, seeds are rarely planted immediately after harvest. Early studies in cotton described an increase in germination when cotton seeds were submitted to air-drying and short-term storage before planting in comparison to freshly harvested seeds. [65]. Although it may seem disadvantageous in agricultural crops, at a first glance, for fresh seeds to halt germination, this adaptive mechanism could prevent a circumstance called preharvest sprouting in which seeds prematurely germinate while still attached to the parental plant causing tremendous economic loss [22].

## 2. Our Program

The greenhouse complex initiative at the Texas A&M AgriLife Research and Extension Center in Lubbock, TX was conceived in 1994 to characterize different species in the *Gossypium* genus. The current greenhouse facility was built in 2002 to provide adequate space for germinating, increasing, documenting, and characterizing cotton specimens from the U.S. National Cotton Germplasm Collection. The main objective was to identify traits that could be useful in academic cooperative breeding and molecular projects and breed for complex native traits to complement industry plant-based biotechnology solutions. Our program screened seeds from species of different parts of the world, including landraces, exotic, and wild species [66]. Due to limited seed quantities and other factors such as dormancy or species not acclimated to our environment, optimizations in seed germination had to take place. The program relied initially on previous methodologies and protocols for seed germinating, regenerating, breaking dormancy, etc. [67,68], making adjustments over time to our needs. Between 2005 and 2013, over 800 accessions from the cotton germplasm collection were increased and screened by our program. Several *Gossypium* species were documented and characterized, including wild and exotic species, like *Gossypium thurberi*, *Gossypium aridum*, *Gossypium sturtianum,* among many others. A list of all screened species can be found in Percy et al. [69].

Besides screening germplasm accessions, the greenhouse complex and the specimens characterized were used for creating research hybrid populations originated from interspecific crosses, producing progenitor species seed for cooperative basic genomic studies, and for exhibitions of exotic cotton plants to national and international visitors. 

The construction of this facility also enabled our program to serve the general public. Requests for germinating and characterizing cotton seeds collected in unexpected places were recurrent. Sometimes, the seeds were so old or damaged that germination was not possible. Other times, with a little patience, research, and creativity, seeds were successfully germinated and characterized. 

Today, although there are occasional requests for cotton seed characterization and increases from the general public and academia, our focus has shifted toward using the greenhouse complex as an extension of our field-based cotton breeding program, and the option of a controlled environment for student projects. The following stories, reports and techniques are based on more than 20 years of trial-and-error to obtain successful methods to germinate the seeds of cotton genetic resources for subsequent experimentation and breeding in Lubbock, TX.

### 2.1. Seed Germination In-House Protocols

Seed quantities produced and received by our lab, either from our own program, germplasm collections, or other sources, are usually small and a well-planned germination strategy is critical. Seeds produced in our greenhouse are harvested and ginned in an electric 10-saw tabletop gin with a lint collector. Fuzzy seeds are first delinted, followed by removal of dead, damaged, or immature seeds determined by visual inspection and by the firmness of the seed [67]. Acid scarification can improve cotton seed germination according to our experience and other authors [39,40]. Therefore, all seeds are acid delinted prior to any germination attempt. Our small batch delinting methodology steps are described in Table 1. 

After seeds are dried and placed in labeled card-stock paper envelopes, they are stored in cold storage until it is time to plant. If seeds were freshly harvested and germination request is urgent, delinted seeds are given a cold treatment (stratification method) by placing them in a conventional household freezer at −18 °C for 2 to 5 days. This is our standard method to break primary dormancy when, for some reason, we need to plant immediately after harvest (greenhouse use only). Otherwise, delinted seeds are placed in our on-site cold storage until time for germination (see seed storage section). 

Soaking seeds in hot water could significantly improve germination. Walhood [35] reported remarkably faster and higher germination rates in three cotton species (dormant or not) after hot water treatment. Then, all seeds that will be grown in the greenhouse are soaked in hot water prior to any germination attempt. Seeds are placed in perforated plastic containers and submerged into an 80 °C hot water bath (Precision 180 series, SN. 698091449; Jouan, Incorporation, Winchester, VA, USA) for 90 s and planted immediately after in potted soil. For the hot water bath containers, we simply perforated plastic pill cases using a household drill, so we can keep seeds from different sources labeled and separated.

If there is no germination after 15 days, and seeds are not dead (soft or rotten), seeds are deemed dormant. Our program has tested multiple germination strategies for breaking dormancy over the years, particularly in the early days when exotic and wild types were common on our work bench. Here are some of the methods we have tried in our lab that yielded positive results.

#### 2.1.1. Seed Coat Mechanical Scarification

When dealing with hard seeds, a mechanical rupture of the seed coat is sometimes effective to increase water absorption and break physical dormancy [30,38]. Among the tools used for this type of scarification are garden clippers, wire cutters, emery board files, and a Dremel grinder (for smaller seeds). A small cut is cautiously made on the seed with a cutter, or part of the seed coat is removed with a grinder. 

#### 2.1.2. Complete Removal of Seed Coat 

We have also tried the complete removal of the seed coat to isolate the embryo. Removal of the seed coat to facilitate and hasten germination is performed in a multitude of crop species, especially fruit crops with solid and sizeable seeds [70,71,72,73]. Studies demonstrated that removing the seed coat of cotton seeds can enable and accelerate germination especially when dealing with hard seed. Simpson et al. [27] reported complete germination after 2 days in embryos that seed coats have been removed, independent of the varietal tendency of late germination. In a recent study, Wang et al. reported 7% early germination in intact freshly harvested cotton seeds versus 82% germination in isolated embryos [74]. Since cotton has a much smaller seed than peaches and mangoes, for example, removing its seed coat without damaging the embryo is a meticulous, almost surgical job. A small cutter is used to carefully remove the seed coat. The delicate embryo is then placed in a germinator (30 °C, 85% relative humidity) on a petri dish with water agar.

#### 2.1.3. Petri Dish with Water Agar

**Figure 2:**
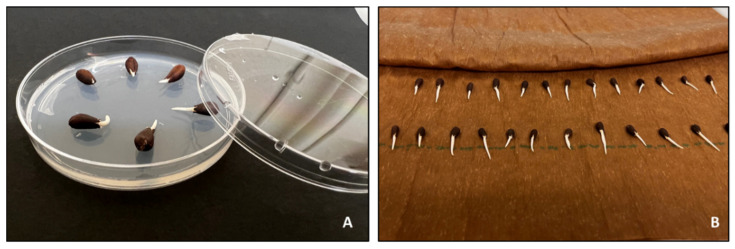
Cotton seed germination in water agar (**A**) versus rolled germinating paper (**B**).

Germinating seeds in a petri dish with water agar substrate is an alternative to using germinating paper (Figure 2). Agar substrate is often chosen over the rolled germinating paper in our lab for its convenience on daily checks of germination rate (clear Petri dishes, instead of unrolling and rolling paper), particularly for species with a small, delicate seed. Water agar (2%) is a stiff gelatinous solution made of a mixture of agar and distilled water [75]. Seeds are placed on top of the agar substrate and placed in a germinator (30 °C, 85% relative humidity). Seeds are checked for signs of germination from day 3 to day 15. When seeds germinate, the substrate is discarded, and germinated seeds are transplanted into potted soil.

#### 2.1.4. Gibberellic Acid

Gibberellins are known to play an important role in a variety of physiological processes, including seed germination [74,76,77]. Seed treatment with gibberellic acid (GA_3_) for inducing germination is rarely used in our lab because in our case, we have not obtained significantly higher germination efficacy with this method. However, on a few occasions, the method described by Rao et al. [67] was attempted. Germination towels are soaked in a solution of 0.05% GA_3_, seeds are placed on the soaked towels, rolled, and placed in the germinator (30 °C, 85% relative humidity) for a maximum of 15 days. If the seeds were known to have a more resistant, impermeable coat, prior scarification, or removal of the seed coat would be performed. 

#### 2.1.5. Smoke

The most unusual method attempted by our lab to break cotton seed dormancy is using smoke. Among the accessions characterized from the U.S. National Cotton Germplasm Collection were Australian wild cotton species. Our conventional germination methods such as hot water treatment, scarification, and planting in water agar were unsuccessful, particularly with *Gossypium sturtianum*. Investigating the typical environment in which the species is grown, researchers noted that in several Australian native flora, grass fires and smoke are a cue for germination [78,79,80,81]. Moreover, compounds in smoke from burning plant material have been previously identified as germination-promoting and have been the subject of several germination studies in agricultural and horticultural species [82,83,84,85]. There was (or is), to the best of our knowledge, no scientific evidence of breaking dormancy of cotton seed with smoke. However, as Tieu et al. stated, dormancy and germination responses in different Australian native plant species are complex and not uniform, and the way a species reacts to environmental conditions is not specific to genera, seed type, or any group category [79]. Therefore, it was worth a try. A simple experiment was designed where *Gossypium sturtianum* seeds were placed on an elevated tray in a clear plastic box. A small fire was started using dry plant material and then extinguished to produce smoke. The box was sealed, and the seeds were left in the closed container with smoke for 20 min. Seeds were then plated in water agar and placed in a germinator for 15 days. The germination success rate was approximately 20% compared to the previous 0%. Interestingly, new seeds derived from those plants had excellent germination and did not require any other treatments other than the habitual delinting + hot water bath. Whether or not that smoke process could be replicated, remains to be answered.

Following are further considerations when dealing with seed germination on a laboratory/greenhouse scale.

#### 2.1.6. Seed Storage

For seeds that do not need to be planted immediately or seeds that will be part of the program seed stock, after delinting and drying, cotton seeds are placed in a labeled 5 × 8 trifold seed envelope. The envelopes are stored in labeled wooden drawers in cold storage. We maintain our cold storage at around 5 °C and approximately 30% humidity with the aid of de-humidifiers. 

#### 2.1.7. Photoperiodism

Many seeds received from germplasm collections are exotic to our region and therefore, are not adapted to our environment. *Gossypium raimondii*, for example, is daylength sensitive and requires special treatment during its seed increase. The greenhouse strategy for germinating and growing *Gossypium raimondii* is to schedule planting for late October/early November. This takes advantage of the natural day and night ratio for this time of the year in West Texas, meeting the requirements of the species to initiate its reproductive stage. However, for special time-sensitive projects requiring, for example, cross-pollination of daylength neutral and sensitive plants, greenhouse blackout covers may be necessary to limit daylight hours, “tricking” the plant into reproductive growth. We have used small black-out covers attached to garage door openers on a timer to automate the process.

## 3. Appendix-Stories

On one occasion, seeds from wild cotton plants growing on an abandoned cattle ranch in the Turks and Caicos Islands were sent to us. This ranch had previously been a cotton plantation at the end of the 18th century where Loyalist refugees settled after the American Independence War. History tells us these refugees established plantations on the islands to grow cotton for export using the Anguilla variety, commonly known as Sea Island Cotton. According to Fryxell [23], Sea Island cotton was developed around that time. The farmers harvested bat guano in caves found throughout the island to use as a source of organic fertilizer. The plantations lasted for approximately 30 years before soil exhaustion, drought, insects, and the War of 1812 destroyed them (information collected from Turks and Caicos tourist guides). The seeds sent to our lab were collected close to one of the bat caves from isolated wild cotton plants, likely heirloom remnants from cotton varieties grown in that region more than 200 years ago. Sea Island cotton is a *Gossypium barbadense* species type that was greatly valued for its high fiber quality until the middle of the 20th century here in the United States. It was produced mainly in the coastal and offshore areas (lowlands) of Georgia and South Carolina [86]. The seeds we received were successfully germinated, and plants were grown in the greenhouse for exhibitions to students and visiting scientists.

On another occasion, old cotton seeds were found in an abandoned Texas gin dated from the early 1900s. The building was slated for demolition and the seeds were sent to our program for regeneration. The seeds were so damaged that regeneration was not possible in that case. Another unsuccessful example was when we received cotton seeds collected in an area where the Caddo Native Americans once grew cotton for textile. The seeds were found in an old glass jar of pimento cheese in an abandoned barn. The lab received only seven seeds that apparently looked undamaged, but despite all the efforts, we were unable to germinate the seeds. In this case, some of the attempted methods included stratification followed by hot water treatment, seed coat scarification, and seed coat removal followed by water agar plating. 

**Figure 3:**
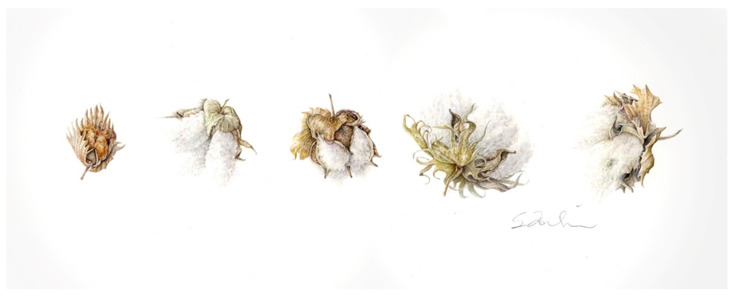
The Evolution of Cotton. Watercolor painting by Lubbock local artist Susan Tomlinson. From left to right: *Gossypiodes kirkii, Gossypium arboreum, Gossypium herbaceum, Gossypium hirsutum, Gossypium barbadense.* Watercolor on paper, 7 × 16 inches. ©2020 Susan Tomlinson.

More recently, Susan Tomlinson, a local artist, reached out to us in search of some unusual cotton species to use as models for botanical illustrations that will be in an upcoming international exhibition. We had growing in our greenhouse a specimen of *Gossypioides kirkii*, progenitor species belonging to the same tribe as the genus *Gossypium* [87]. She sketched and painted the bolls of that plant along with other cotton species such as the cultivated *Gossypium arboreum*, *Gossypium herbaceum*, *Gossypium hirsutum*, and *Gossypium barbadense*. Under Leslie Wells’ guidance, our greenhouse manager, she was also able to successfully germinate seeds from *Gossypium nelsonii*, *Gossypium thurberi*, and *Gossypium aridum*. Those plants were grown in the greenhouse, and she is currently preparing botanical illustrations for the exhibition. Below is an example of one of her beautiful watercolor paintings (Figure 3).

## 4. Conclusions

Before plant genetic resources can be utilized in crop improvement, adequate quantities of quality seed must be available. Seeds are living organisms and as such respond to biotic and abiotic factors over time. Germinating and multiplying cotton seeds for research, characterization, or breeding can be a challenge even on a small scale and under controlled environmental conditions. Much of these challenges can be attributed to different requirements based on species, origin, and age, for example. Many tactics can be employed to generate and maintain appropriate research seed stocks, with the amount of required effort linked to seed origin and condition, interest, and priority of research. 

## Figures and Tables

**Table 1:** Delinting process steps at Texas A&M AgriLife Research Cotton Breeding Program in Lubbock, TX.

Steps	Description
1	Fuzzy cotton seeds are placed in a labeled perforated plastic container.
2	The container is submerged in a bucket with 93% sulfuric acid and seeds are stirred for 2–3 min using a wood stake. The friction while stirring the seeds facilitates the delinting process.
3	Seeds are rinsed with tap water to remove the excess sulfuric acid.
4	The container is submerged in a sodium carbonate solution to neutralize the acid.
5	Seeds are rinsed with tap water.
6	Seeds are submerged in methyl alcohol for a few seconds. This step helps with the drying process.
7	Seeds are spread in a screened tray and are placed in the drier.

## Disclaimer

The intention of this paper is to give ideas on what to try if you have a limited amount of exotic/wild/hard cotton seeds and need to make sure you get at least a few seeds to germinate. Sometimes, a single germinated seed is sufficient for the continuation of a project. We are not presenting repeatable research experiments (i.e., treatment versus control) as in a research article, but condensing in one place all germination techniques we have tried throughout the years that yielded positive results. However, results are dependent on seed condition, age, and species. It is a trial-and-error process that we thought would be interesting to share with the cotton community.
